# A Facile Method to Prepare a Hydrophilic/Hydrophobic Metal Surface by Peptide

**DOI:** 10.3390/ma11081289

**Published:** 2018-07-25

**Authors:** Chunying Ma, Chengqing Yuan, Pan Cao

**Affiliations:** 1 School of Energy and Power Engineering, Wuhan University of Technology, Wuhan 430063, China; mying2003@126.com (C.M.); mying2003@163.com (P.C.); 2 School of Materials Science and Engineering, East China Jiaotong University, Nanchang 330013, China

**Keywords:** facile method, peptide, modification, hydrophobic/hydrophilic, optimization

## Abstract

A facile method to prepare a hydrophilic/hydrophobic metal surface by metal-binding peptide was proposed in this article. Metal-binding peptide sequenced NLNPNTASAMHV was taken as the target peptide to interact with stainless steel. The surface morphology, roughness and Fourier-Transform Infrared spectroscopy (FTIR) showed that some changes occurred on the modified stainless steel surface. Not only were the surfaces coarser but also some organic groups appeared on the modified sample surfaces. By comparing the CAs of all the samples, the most suitable concentration of peptide and treating time were determined. A new and facile way to endow some metals surfaces with hydrophobicity or hydrophilicity has been developed, which is useful especially for antibiofouling.

## 1. Introduction

As common metal, stainless steel is widely used in food, milk, medical, marine and the oil industry etc., yet it cannot be free from bacterial attachment [1] and corrosion. The corrosion cannot always be separated from the role of the microorganisms, thus the stainless steel has attracted many researchers to attempt to improve its antifouling properties. The corrosion damage initiated or aggravated by microorganisms is called microbiologically influenced corrosion (MIC) and is a part of biofouling [2,3,4]. The MIC will bring huge economic losses. For example, for a ship, after the sea bacteria binds to and grows on the hull, not only will the overall weight of the ship increase but also its surface will get coarser. These are disadvantageous and will eventually reduce the ship’s service life. If the bacteria are bound to the bottom of the ship, the fluid dynamics resistance will increase, and the fuel consumption will increase as well [5,6,7,8,9,10,11,12]. The MIC is an important limitation of the development of the shipping industry [13]. The fouling of microorganisms can corrode almost all of a metallic object’s surface. The lost cost of biofouling may be in the billions of dollars annually worldwide [14], and that is why there is an outstanding interest in the development of effective and economical control measures.

Methods dealing with biofouling [15,16] were developed a long time ago; mechanical detachment, use of chemicals, and surface modification are the most common methods. Mechanical detachment is a stripping method where the microbiological organisms are stripped by some mechanical means after being corroded. These methods are very expensive and time consuming and are difficult to realize because their efficiency is low. Using chemicals is widely adopted in the present, this method aims to deactivate or kill the biofouling organisms. As these chemicals often contain toxic components or heavy metals, they will irreversibly damage the biological chain. Most of these methods are environment destroying. The other shortcoming of using chemicals is that the validity duration is short and repainting is necessary after a period of time. Therefore, the third method of surface modification is becoming more and more popular. It aims at gaining a low or non-sticking surface by altering the surface chemical composition, morphology, or roughness to endow it with hydrophobicity [17]. As we know from Young’s equation, when the contact angle (CA) of a surface is more than 90°, the surface is hydrophobic and holds a low surface energy. Comparing with the other methods, this method is environmentally friendly [18] and has a long period of validity. However, some of them need special devices and technologies; it is not a very economical method and difficult to implement. Studies showed that peptides are efficient at binding to some metals, such as gold [19,20,21], silver [22,23], platinum [10,24], titanium [25], cadmium and mercury [9], zinc oxide [26,27], mild steel and aluminum [11,28,29], and so on. Peptide may be a useful tool to modify metals that will be an avirulent, persistent, self-repairing activity for the peptide. 

The GRAVY (Grand Average of Hydropathy) value for a peptide or protein is calculated as the sum of the hydropathy values of all the amino acids divided by the number of residues in the sequence. The greater the negative, the more hydrophilic the peptide. In this study, we took stainless steel as the experimental metal to interact with peptide sequenced as NLNPNTASAMHV [11], and the GRAVY is negative. We measured the CAs of all the samples and found that the modified surfaces that the peptide used were also hydrophilic. Based on this result, we will conduct further study on seeking or designing suitable peptides to endow the surface with hydrophobicity and the ability to antibiofoul. It will be preferable in many industries.

## 2. Materials and Methods

The metal coupons used in this binding assay were grade 304 stainless steel plates with a diameter of 10 mm and a thickness of 1 mm. The coupons were annealed at 1040 °C for 1 h to release stress before further treatment.

Before being treated by organic peptide, the coupons were polished sequentially by sandpaper grit up to 1200 and finally polished with an aqueous slurry of 0.05 μm colloidal silica to achieve a uniform surface roughness. Then the coupons were cleaned thoroughly with dish detergent, distilled water, and ethanol successively. After being immersed in 95% (*v*/*v*) ethanol for 15 min with gentle agitation, the coupons were rinsed with distilled water again, and then washed with acetone for 1 min. The coupons were then rinsed thoroughly with distilled water and dried in Biological Clean Room for future use.

Peptide sequenced NLNPNTASAMHV was synthesized independently by solid phase peptide synthesis and purified by Shanghai Science Peptide Biological Technology Co., Ltd. (Shanghai, China), whose molecular formula, sequence, and other properties were shown in Table 1. Peptide solutions were prepared separately for different concentrations, 5 μg/mL, 10 μg/mL, 20 μg/mL, 40 μg/mL, and 100 μg/mL with sterile phosphate buffered saline (PBS, pH 7.4).

**Figure 1:**
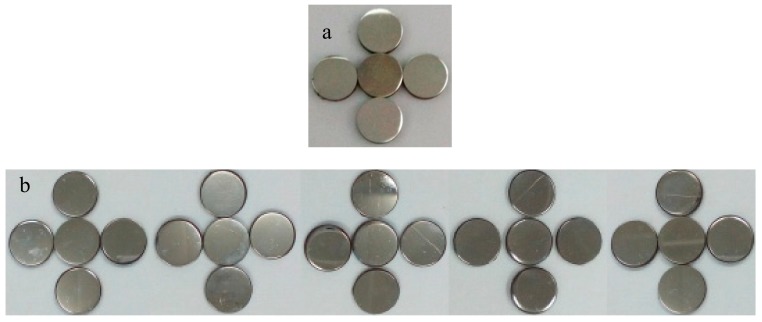
Surface images of the coupons before and after the peptide treatment. (From left to right are coupons treated with different concentrations: 5 μg/mL, 10 μg/mL, 20 μg/mL, 40 μg/mL, 100 μg/mL; each concentration has five samples, which were treated for different times: 40 min, 60 min, 80 min, 100 min, and 120 min). (**a**) were the original stainless steel coupons; (**b**) were all the modified stainless steel coupons.

The prepared coupons were placed respectively in 6-well tissue culture plates (1 coupon per well) and covered with sterile phosphate buffered saline (3 mL per well) containing peptide with different concentrations as mentioned above. The tissue culture plates encasing the coupons were placed into a shaker with continuous agitation at room temperature (RT) for several different times: 40 min, 60 min, 80 min, 100 min, and 120 min. The coupons were then washed repeatedly with distilled water to remove the unbound peptide, then allowed to dry in air at room temperature. All coupons were shown in Figure 1.

Scanning Electron Microscope (SEM, JSM6300, JOEL, Kyoto, Japan) was used to observe all coupons. The lateral resolutions for SEM were less than 3.0 nm. The accelerating voltage is 15 kV. Five different locations on each coupons were scanned and multiple images were collected.

We further obtained the roughness *S_a_* of the samples from the Atomic Force Microscope (AFM; Digital Instruments Dimension XE-70, Park Systems, Suwon, Korea) images through software. The AFM was in non-contact tapping mode. *S_a_* shows the average roughness of the surface. The bigger the value of the *S_a_* is, the coarser the surface gets. *S_a_* is defined as follow:

(1) $$S_{a}=\frac{1}{MN}\sum_{k=0}^{M-1}\sum_{l=0}^{N-1}\left|z\left(x_{k},y_{l}\right)-\mu \right|\;$$

where *μ* is the mean height:

(2) $$\mu =\frac{1}{MN}\sum_{k=0}^{M-1}\sum_{l=0}^{N-1}z\left(x_{k},y_{l}\right)\;$$

(3) $$S_{q}=\sqrt{\frac{1}{MN}\sum_{k=0}^{M-1}\sum_{l=0}^{N-1}{\left(z\left(x_{k}-y_{l}\right)-u\right)}^{2}}\;$$

While *S_ku_* shows the height distribution kurtosis of the surface, and is to describe the amplitude distribution curve for the points of the surface profile. The topography height of the surface will be in the center of the base plane when the value of *S_ku_* is greater than 3.0. It is better that the value of *S_ku_* is closer to three, which means that the surface is smoother. *S_ku_* is defined as:

(4) $$S_{ku}=\frac{1}{MNSq^{4}}{\sum_{k=0}^{M-1}\sum_{l=0}^{N-1}\left(z\left(x_{k}-y_{l}\right)-\mu \right)}^{4}\;$$

In the above equations, *M* is the collected points in the *x* direction of the reference plane, *N* is the collected points in the *y* direction of the reference plane, and *S_q_* is the surface root mean square deviation.

Fourier-Transform Infrared Spectroscopy (FTIR; Nicolet AVATAR360, Nicolet, Madison, AL, USA) was used to determine whether there were organic groups on the surfaces and their categories. The spectra ranged from 4000 to 500 cm^−1^ with resolution of 0.09 cm^−1^ and have been collected using the infrared spectrometer equipped with the attenuated total reflection (ATR) accessory. 

The surfaces were also analyzed using a contact-angle meter (CA, JC2000D1, Zhongchen, Shanghai, China). We got the static water contact angles of the sample surfaces based on the sessile drop method. All contact angles were determined by averaging five different point values measured on each surface.

## 3. Results

### 3.1. Surface Topography and Roughness

**Figure 2:**
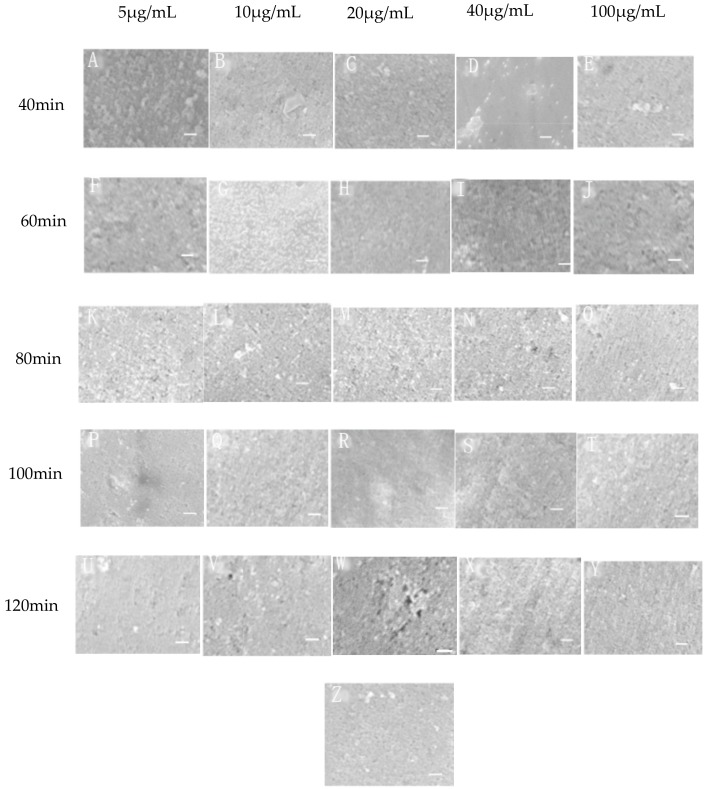
Scanning Electron Microscope (SEM) images of the coupons (10 K). (**A**–**Y** were respectively at different concentration and time; **Z** was the original metal; Scale bars in all photos represent 5 μm).

From the SEM images (Figure 2) we could see that the surface of the original metal was different from the modified samples in appearance. Most of the modified samples were coarser and when the incubating time was 80 min the surfaces became even coarser. It showed that there were some changes occurring on the metal surfaces in the solutions of peptide.

**Figure 3:**
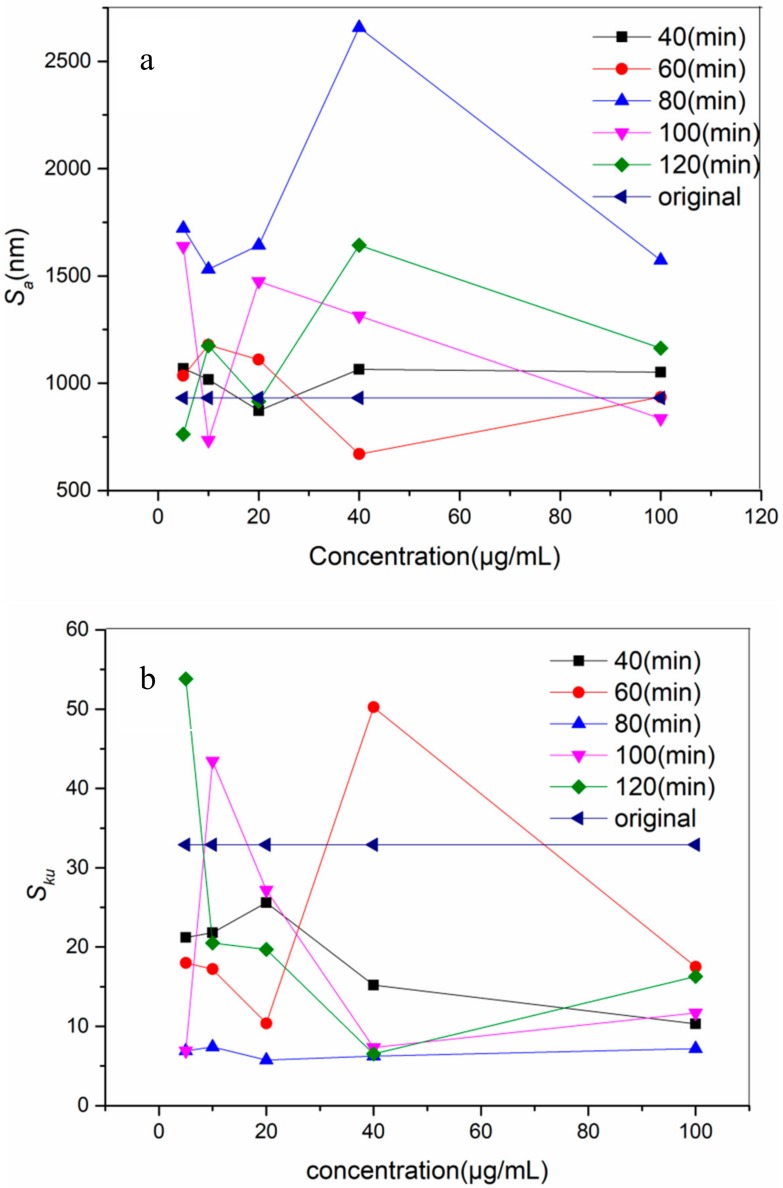
Roughness of the modified metals: (**a**) showed *S_a_*; (**b**) displayed *S_ku_*.in different conditions.

The samples were further observed using AFM to obtain the roughness data of the metals from the AFM images. The roughness of the original metal was *S_a_* = 931 nm, *S_ku_* = 32.9, and the roughness of the modified coupons was demonstrated in Figure 3. It showed that at different times and concentrations, *S_a_* and *S_ku_* were variable, while those of the original metal was constant. It confirmed the result of the SEM that there was some difference between the original metal and the modified ones in *S_a_* and *S_ku_*. When the metals were incubated for 80 min, the surfaces turned the coarsest, independent of the concentrations (Figure 3). It also showed that with the increase of the concentration, the roughness of the surface was different. When it was 10 μg/mL, the value of the roughness was the lowest, then it went up again till 40 μg/mL, the value of the roughness was the highest. Comparing with the original metal, all the modified surfaces had a higher roughness. As we know, *S_ku_* shows the height distribution kurtosis of the surface, and it is better when the value of *S_ku_* is close to three. From Figure 3 we can see that, at 80 min all the values of *S_ku_* were closer to three (Figure 3b). thus 80 min was the most suitable incubating time. We can bring out from above data that the surfaces of the metal were different from the original one, and these differences were most distinct at 80 min, with concentrations of 10 μg/mL and 40 μg/mL. Therefore these two concentrations deserve extra attention.

### 3.2. FTIR Analysis

**Figure 4:**
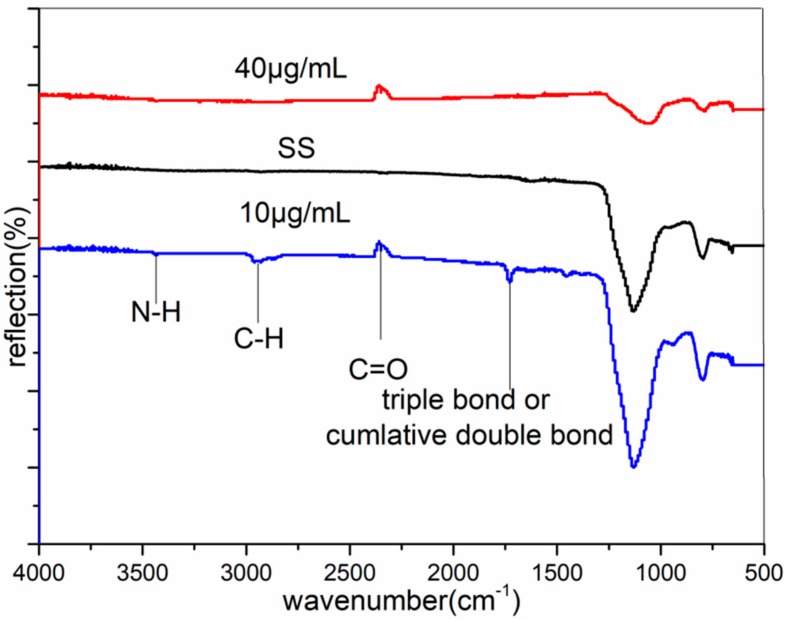
Fourier-Transform Infrared Spectroscopy (FTIR) of the original stainless steel and the stainless steel of 80 min, with concentrations of 10 μg/mL and 40 μg/mL. SS was the FTIR of the original stainless steel.

Due to its sensitivity to the chemical composition, FTIR is one of the classical methods for the structural determination of small molecules [30]. To figure out what happen on the coupons surface, we paid additional attention to coupons treated at 80 min with concentrations of 10 μg/mL and 40 μg/mL. Their FTIR results are shown in Figure 4. By comparison, we can see that there were three obvious peaks in it. The peak that appeared around 2800–3000 cm^−1^ [29,31] was corresponding to saturated C-H stretching vibration. It should be alkane. The peak that occurred around 1700 cm^−1^ [29,31] was in the carbonyl group region, which was due to C=O stretching. While a strong peak at 2100–2400 cm^−1^ [29,31] showed the presence of a triple bond or a cumulative double bond. The peak near 3300–3500 cm^−1^ [29,31] showed the presence of N-H. At a concentration of 40 μg/mL, only the peak that appeared at 2100–2400 cm^−1^ was left and was nearly the same with a concentration of 10 μg/mL. That means some organic groups disappeared. It is probably because of the gathering of the peptide, where the peptide binding to the metal decreased or was unevenly bound to it and was washed-out in later rinsing. This also was the reason that the surface of concentration of 40 μg/mL was non-uniform and coarser than the concentration of 10 μg/mL.

### 3.3. Measurement of Water Contact Angle

**Figure 5:**
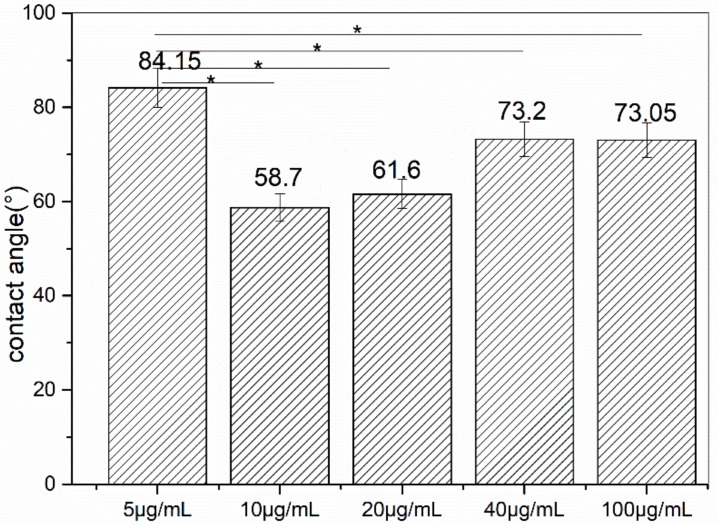
Contact angle of the modified steels (80 min), * *p* < 0.05.

The contact angle indicates the hydrophilicity of a solid surface and it can to some extent show the surface energy of a solid material [32]. A material with a low surface energy will benefit from many domains and is popular in industrial and marine antifouling for its stability in liquid medium and ability to reduce the interaction with the active cell. The higher the surface energy is, the easier the solid surface is to be adhered, and further the heavier the corrosion. To see if the modified stainless steel has a different hydrophilicity than the original one, and the relationship between the hydrophilicity of the metal and that of the peptide used, we measured the contact angles of the coupons. Five different points were picked from each sample to measure the contact angles of the surface and the average contact angles were calculated (Figure 5). The contact angle of the original metal is 84°.

The water contact angles of the modified metal surfaces (80 min) almost all decreased compared to the original one, except for the value at a concentration of 5 μg/mL. All the other values were smaller than the original one, and when the concentration was 10 μg/mL, the value was the lowest. Then with the increasing of the concentration, CA got bigger again. After arriving at 40 μg/mL, the value was almost the same. The result indicated that the peptide treatment changed the metals affinity to water; that is to say, there were some changes that occurred during the process of modification. And at the beginning, the number of peptides on the metals was not enough to cover the whole surface of the sample, so these changes did not occur distinctly. When the concentration became greater, this difference became significant. From the GRAVY of the peptide, we knew that it was hydrophilic. After reacting with the peptide, the surface of the coupons showed the same hydrophilicity with the peptide. Thus we were challenged to find a suitable functional peptide to interact with the metal, to show that endowing the surface with hydrophobicity is possible.

## 4. Discussion

The difference among the metals in appearance and Sa was not distinct. This is probably because the largest concentration of the peptide solution used in this experiment was 100 μg/mL and the layer of the peptide binding to the metals was too thin. The color of the modified metals was almost the same as the original one (Figure 1). Similarly, no significant changes were observed among the modified metals obtained in different concentrations and incubating times. The SEM images (Figure 2) demonstrated the differences in roughness from vision, but they could not provide definite proof. Sa was gained based on the AFM images by software. It made it certain that after the treatment some changes happened. Figure 3 showed that, at 80 min, different concentrations and the roughness of surfaces were all obviously bigger than that of the other times. In other words, under this condition, the peptide bonds to the metal surface strongly and uniformly. It was consistent with the result of FTIR and CA. Thus an incubating time of 80 min became the suitable interaction time. At 80 min, when the concentration was 10 μg/mL, the Sa and Sku were the smallest and the Sku was close to three, that is to say the surface treated at this condition was smoother and more uniform. This is probably because at a concentration of 5 μg/mL, the number of peptides was very few and not enough to cover the whole metal surface and bind to it. From a concentration of 10 μg/mL, with increasing of the concentration, the number of peptides grew. When it was at 10 μg/mL, it attained the biggest value. After that, with the concentrate increasing the peptides binding to the surface were in excess and reunited in some form. The bigger the concentration, the more reunited the peptide. It resulted in the roughness of the surface, which was increasing and then decreasing in some extent. When the time was less than 80 min, the peptide could not react with the metal sufficiently. After 80 min, some peptide would get together and decrease the roughness of the surface. Therefore, when the condition time was 80 min with a concentration of 10 μg/mL; the surface was the most uniform one.

Biofouling is related to the formation of biofilm. The formation of a stable biofilm on the surface of materials by microorganisms is divided into several main stages, of which attachment is the prerequisite. The hydrophobic surface is not easily attached to by microorganisms, or even when it does attach, the adhesion is weak. They tend to be removed under an external force, such as water flow. Hydrophilic surfaces are more prone to adhesion. After the metal-binding peptide reacts with the metal, the contact angle of the material surface changes, that is, the hydrophilicity/hydrophobicity changes, so the possibility of biofouling also changed accordingly.

At 80 min, a concentration of 5 μg/mL of the value of CA was almost the same as the original metal (Figure 5), while at other concentrations, the values were smaller. At a concentration of 10 μg/mL, at 80 min, this difference became the greatest. This confirmed that under these circumstances the peptide interacted with the metal more successfully and stably, and thus the properties of the surface were distinctly changed. The organics binding to the surface should be the reason the changes occurred on the surface.

## 5. Conclusions

The following conclusions can be drawn according to the above analysis.

(1) A metal binding-peptide could modify the metal surface properties.
(2) The optimal incubating time and concentration was 80 min and 10 μg/mL.
(3) The modified metal has the same hydrophobicity with the metal binding-peptide.

This modification process was facile and avirulent, persistent, and self-repairing for the peptide activity. We believe that the method developed in this study is a useful tool to modify metals and endow them with hydrophobicity, which is meaningful in antibifouling for many fields.

## Figures and Tables

**Table 1:** The bioinformation of the peptide used in assay.

Properties	Description
Amino acid sequence and number	NLNPNTASAMHV 12
Aliphatic index	73.33
Isoelectric point (pI)	6.74
Molecular Weight	1268.4
GRAVY	−0.275
formula	C52H85N17O18S1
